# Adenosine-Related Mechanisms in Non-Adenosine Receptor Drugs

**DOI:** 10.3390/cells9040956

**Published:** 2020-04-13

**Authors:** Kenneth A. Jacobson, Marc L. Reitman

**Affiliations:** 1 Molecular Recognition Section, Laboratory of Bioorganic Chemistry, National Institute of Diabetes and Digestive and Kidney Diseases, Bethesda, MD 20892, USA; 2 Diabetes, Endocrinology and Obesity Branch, National Institute of Diabetes and Digestive and Kidney Diseases, NIH, Bethesda, MD 20892, USA; marc.reitman@nih.gov

**Keywords:** adenosine receptor, nucleoside transport, CNS, inflammation, cardiovascular system, pain

## Abstract

Many ligands directly target adenosine receptors (ARs). Here we review the effects of noncanonical AR drugs on adenosinergic signaling. Non-AR mechanisms include raising adenosine levels by inhibiting adenosine transport (e.g., ticagrelor, ethanol, and cannabidiol), affecting intracellular metabolic pathways (e.g., methotrexate, nicotinamide riboside, salicylate, and 5-aminoimidazole-4-carboxamide riboside), or undetermined means (e.g., acupuncture). However, other compounds bind ARs in addition to their canonical ‘on-target’ activity (e.g., mefloquine). The strength of experimental support for an adenosine-related role in a drug’s effects varies widely. AR knockout mice are the ‘gold standard’ method for investigating an AR role, but few drugs have been tested on these mice. Given the interest in AR modulation for treatment of cancer, CNS, immune, metabolic, cardiovascular, and musculoskeletal conditions, it is informative to consider AR and non-AR adenosinergic effects of approved drugs and conventional treatments.

## 1. Introduction

Drugs can affect adenosine signaling in multiple ways. They can act directly as ligands for one or more of the four G protein-coupled receptors (GPCRs) for adenosine [1,2,3]. Alternatively, they can stimulate or inhibit pathways of adenosine generation, degradation, or clearance [4,5,6,7]. Some drugs that were not originally postulated to involve adenosine mechanisms are now suspected or confirmed to be doing so [8,9,10,11]. Here, we review selected examples of adenosinergic mechanisms of action (MoA) proposed for diverse drugs and treatments, emphasizing examples where the compound does not bind directly to an adenosine receptor (AR). On one hand, clear characterization of adenosine-related mechanisms in non-adenosine receptor drugs can potentially lead to repurposing of clinically approved drugs. On the other hand, the strength of the current experimental support varies widely, and additional experiments are needed to validate the role of adenosinergic signaling in the MoA of certain drugs.

Among the many approved pharmaceuticals, pharmacological tools, folk remedies, and compounds with poorly characterized biological activity that have been ascribed an adenosinergic MoA, there are putative agonists and antagonists, as well as compounds that interfere with the formation, degradation, and/or transport of adenosine. Several enzymes that are responsible for the production or metabolism of adenosine also regulate its location and concentration. Intracellular adenosine kinase (ADK) and adenosine deaminase (ADA, two forms, one intracellular and one extracellular) remove adenosine, while extracellular CD73 (5′-nucleotidase; and indirectly CD39) and intracellular S-adenosyl-L-homocysteine hydrolase produce adenosine [1,4,5]. In addition, there are equilibrative (ENT1-3, SLC29 family) and concentrative (CNT1-3, SLC28 family) transporters with various levels of specificity and selectivity [12,13], which, when inhibited, can increase extracellular nucleosides, including adenosine. Thus, in addition to the ARs, we need to consider the role of nucleotidases, phosphatases, and other enzymes that produce or catabolize adenosine, and the role of nucleoside transporters on the putative adenosinergic MoA of these diverse compounds.

### 1.1. Endogenous Adenosine

**Figure 1:**
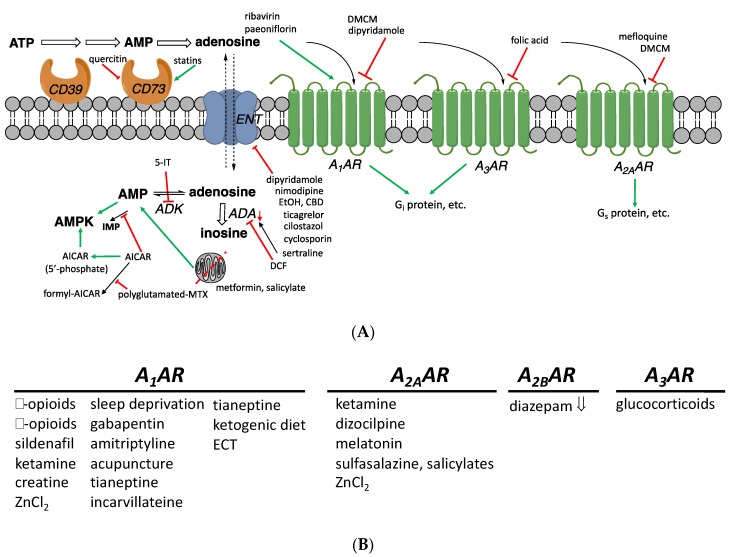
(**A**) Adenosinergic pathways and their putative modulators among non-AR drugs. These interactions have been found in model systems in vitro or in vivo and do not necessarily imply that a compound achieves the indicated effect when administered in humans. Intracellular processes resulting in elevated adenosine concentrations are shown. The A_2B_AR (not shown) activates G_s_ protein and has a low affinity for adenosine. (**B**) Drugs or treatments that influence adenosinergic signaling, including those having an undetermined or unclear mechanism (all are stimulatory, except diazepam). Inhibitory and stimulatory effects on the production of cAMP, mediated by Gi and Gs proteins, are shown. Intracellular adenosine concentrations can also be raised through inhibition of ADK (e.g., by 5-iodotubercidin (5-IT, structure not shown), ABT-702 **18**, or A-134974 **19**) or of adenosine deaminases (e.g., by deoxycoformycin, DCF **20**). Moreover, AICAR inhibits an enzyme that metabolizes AMP by deamination, to indirectly increase intracellular adenosine. Compounds that are reported to influence adenosine signaling, but through undetermined AR subtypes, are not listed here.

Extracellular adenosine is considered a “retaliatory metabolite” in a protective feedback control pathway against excessive intracellular ATP consumption [14]. Intracellular AMP and adenosine concentrations rise when ATP is depleted in low energy states and function as metabolic stress signals. These stress signals then lead to activation of non-AR-dependent mechanisms, such as AMP-dependent protein kinase (AMPK) [15] and, indirectly, to AR activation by extracellular adenosine. Extracellular adenosine can also be produced locally during injury or an inflammatory response [3]. Adenosine has been identified as an anti-inflammatory mediator and an endogenous antiseizure substance in the brain [5]. Some pathological states are associated with an imbalance in adenosinergic signaling. In cancer, local adenosine levels are elevated in the tumor microenvironment and allow a tumor to evade immune attack, suggesting the potential of targeted pharmacological blockade of adenosinergic signaling for use in cancer therapy [16]. Conversely, in other disease states, the removal of extracellular adenosine, for example, as an indirect result of the pathological upregulation of intracellular adenosine kinase in the brain, can exacerbate epilepsy [5]. In bone, reduced A_2A_AR or A_2B_AR signaling has a deleterious effect in models of osteo-and rheumatoid arthritis [17,18]. The four AR subtypes (A_1_, A_2A_, A_2B_, and A_3_; Figure 1) are important in the body’s adaptation to stress [1,3], often in the context of increasing local blood flow by vasodilation. However, a knockout (KO) mouse line lacking all four ARs displayed no differences in growth, fertility, metabolism, and thermal regulation, although long-term survival was diminished [2]. This suggested that ARs have a more pronounced role to regulate allostasis, i.e., to restore the stability of an organism in response to challenges or stresses, than homeostasis, which maintains unperturbed physiological parameters. The interplay of generally anti-inflammatory adenosine acting at ARs with related adenine nucleotides acting at pro-inflammatory P2X and P2Y receptors is to be considered in the larger context of purinergic signaling [19].

Plasma adenosine is locally cleared in a few seconds [20]. In humans, pharmacologic doses of intravenous adenosine have a plasma half-life of only ~1 min [21]. These properties mean that plasma adenosine levels (100–1000 nM) [20], while important, are not the only relevant concentration. Adenosine is a local or paracrine modulator, so local levels dictate most of the physiologic effects. This means that adenosine has a plethora of actions, which depend on the particular cells and specific tissue involved.

### 1.2. Action of Various Drugs Involves Adenosine

While the strength of the data varies, adenosine is proposed to be involved in the effects of ethanol, anti-inflammatory drugs, vasodilators, and various drugs and natural products used for treating depression, anxiety, behavioral and sleep disorders, and pain. Possible actions include directly or indirectly modulating adenosine levels, acting directly on ARs or their binding partners (receptor or non-receptor; see Section 2.5).

There is a wide range of pharmacologically active substances that were not originally developed as AR ligands but have a principle or secondary MoA involving extracellular adenosine and ARs. Many substances alter adenosine signaling indirectly, by increasing or decreasing the level of extracellular adenosine [4,8] or by affecting protein partners of the ARs. Often, this modulation of adenosinergic signaling does not involve a measurable, direct binding interaction with the ARs, as determined early on for anxiolytics that nevertheless interact with purinergic pathways [11].

### 1.3. Known AR Ligands

**Figure 2:**
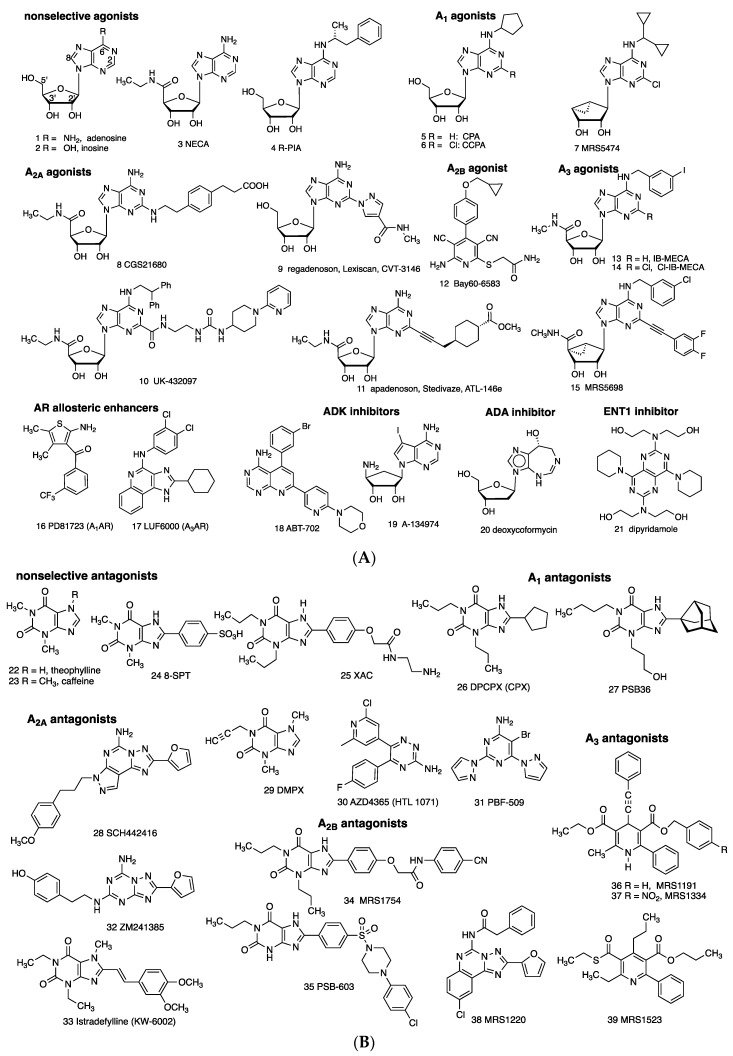
(**A**) Direct (orthosteric agonists **1**–**15**) and indirect modulators (**16**–**21**) of ARs. Compound **2** binds ARs only weakly (>10 µM), but it activated A_3_AR in vivo [2]. Compounds **1**, **9**, **20**, and **21** are in human use. Compounds **13** and **14** are in clinical trials. Agonists **4**, **10**, and **11** were previously in clinical trials. (**B**) Competitive AR antagonists (**22**–**39**). Compounds **22** and **33** are in human use. Compounds **30** and **31** are in clinical trials for cancer.

Directly acting, potent agonists (**1**–**15**) and antagonists (**22**–**39**) for each of the ARs are available as pharmacological probes, and, in several cases, agents approved for human use (Figure 2) [22]. AR agonists **1** (adenosine) and **9** (regadenoson) and are approved for clinical use in myocardial perfusion diagnostics and/or supraventricular tachycardia diagnosis and treatment. A_3_AR agonists **13** and **14** are in clinical trials for autoimmune inflammatory and liver diseases, respectively, and A_2A_AR antagonist **33** (istradefylline) is used for Parkinson’s disease. Selective A_2A_AR antagonists **30** and **31** are in clinical trials for cancer. The binding affinities of selected AR ligands, many of which have been used in the studies described below, are shown in Table 1. XAC **23** can be considered a pan-antagonist of human ARs, and NECA **3** is a pan-agonist.

There are caveats with the use of many AR pharmacological probes. Marked species differences in the affinities and selectivities of agonists/antagonists have been documented, including between species as similar as the rat and mouse [22,26]. In some cases, insufficient attention is given to species differences and differences between binding selectivities and in vivo selectivities, which can lead to misinterpretation of in vivo data. For example, A_1_AR antagonist DPCPX **26**, and to a lesser degree PBS36 **27**, also antagonizes the A_2B_AR [26]. *R*-PIA **4** is a historical A_1_AR/A_3_AR agonist and is superseded by more selective **5**, **6** (also acts at A_3_AR), Cl-ENBA (structure not shown, diastereomeric mixture), and **7** (in mouse) [22,23,24,26]. CGS21680 **8** is more A_2A_AR-selective in rat than in human [26]. A_2A_AR antagonist DMPX **29** is of low A_2A_ selectivity and is superseded by more selective **28**, while **32** is less selective. Bay60-6583 **12** is marginally A_2B_AR-selective and displays variable efficacy, and antagonists **34** and more potent **35** are widely used [26]. MRS5698 **15** is representative of highly selective (≥10,000-fold) A_3_AR-selective agonists in human and rodents [23]. A_3_AR antagonist MRS1220 **38** is A_3_AR-selective in human but not in rat or mouse, where it is A_2A_AR-selective. MRS1523 **39** is a moderately selective rat or mouse A_3_AR antagonist, while **36** and more potent **37** are human A_3_AR-selective. Moreover, AR ligands are sometimes used at concentrations or doses that exceed the limit of their selectivity, e.g., **1** and **23** (500 µM) were used in a study of andrographolide [27], or without considering their biodistribution. For example, peripheral doses high enough to achieve desired brain levels may have off-target peripheral effects. Thus, there is a need to reexamine some of the earlier findings summarized here, using AR knockout (KO) mice or at least more recently reported selective AR ligands.

### 1.4. Known Modulators of Adenosine Pharmacokinetics

Enzyme and transport inhibitors and AR allosteric enhancers that indirectly modulate the levels of AR activation are shown (**16**–**21**; Figure 2A). The ADA inhibitor deoxycoformycin **20**, an anticancer drug, and ENT1/2 inhibitors dipyridamole **21** and dilazep (structure not shown), with cardiovascular indications, are in human use. Inhibitors of ADK (in rat carrageenan-induced paw edema, ED_50_ 0.13 mg/kg **18**, p.o.) [28] and ADA [29] have anti-inflammatory effects. Increasing the level of extracellular adenosine by ADA inhibition ameliorates experiment colitis [4]. Furthermore, low-dose methotrexate (MTX), which is widely used for treating chronic inflammatory disorders, e.g., rheumatoid arthritis and psoriasis, increases adenosine levels [9].

The widely used anti-platelet and vasodilator drug, dipyridamole **21**, which was initially shown to inhibit cyclic nucleotide phosphodiesterases (PDEs), probably has its main effects by ENT1 inhibition, leading to activation of ARs by increasing local extracellular adenosine concentrations [30]. In the brain, ENT1 and ADK are principally expressed in astrocytes, which are a major source of extracellular adenosine via ATP [5,31].

## 2. Proposed Adenosinergic Mechanism of Diverse Drugs and Treatments

**Figure 3:**
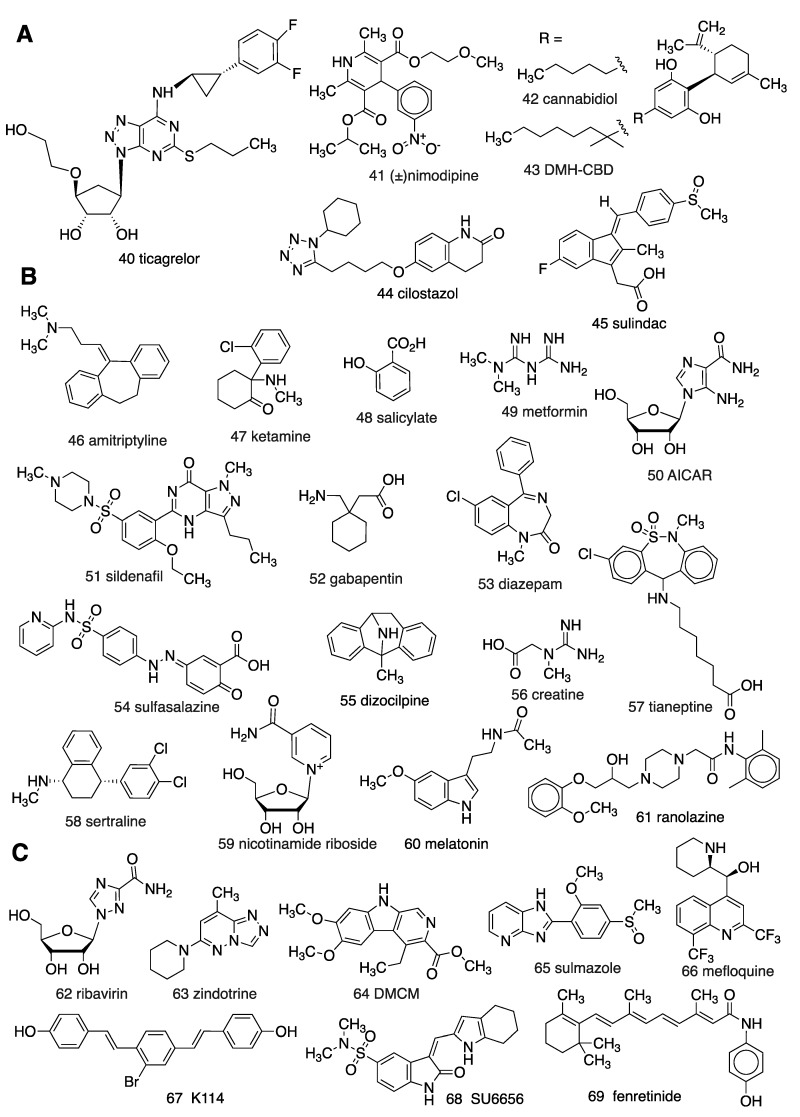
Structures of diverse drugs suggested to have an unanticipated involvement of adenosinergic signaling in their MoA: (**A**) By modulating adenosine transport; (**B**) by unclear mechanisms; (**C**) by direct AR interaction.

Reports dating back nearly four decades implicate adenosinergic mechanisms in the MoA of non-AR drugs (e.g., **40**–**69**; Figure 3). However, the pharmacological tool compounds used in these studies were often ambiguous. The early AR agonists and antagonists were of marginal AR subtype selectivity, and other pharmacological tools, such as CD73 inhibitors, were also suboptimal. Pharmacokinetic considerations are also important. For example, in mice, the highly A_1_AR selective agonist CPA **5**, will activate peripheral A_3_AR at lower intraperitoneal (i.p.) doses than those that activate central A_1_ARs [24]. The majority of these reports do not utilize AR KO mice, so there is a need for re-evaluation of many of these conclusions, using more modern methods. Nevertheless, we present here the evidence as published, acknowledging that some conclusions deserve reinterpretation.

### 2.1. Vasoactive and Other Cardiovascular Effects

Adenosine **1** was first purified in 1929 by Drury and Szent-Györgyi [32], after it was found to cause bradycardia, now known to be an effect of A_1_AR activation in the sinoatrial node. The smooth muscle relaxant and vasodilatory properties of adenosine, by activation of the A_2A_ and A_2B_ARs, led to the early suggestion that adenosine agonists be used as anti-hypertensive agents [33]. Although there were problems with this approach (e.g., side effects and desensitization), other cardiovascular applications of AR agonists (such as antiarrhythmics) and antagonists have been considered. Furthermore, various cardiovascular physiological effects ascribed to presumed non-AR modulators, e.g., ticagrelor **40** (see below), are now known to be mediated by adenosine.

Ischemic preconditioning of the heart, i.e., when short ischemic episodes improve the outcome of a subsequent ischemia event, is thought to involve ARs, consistent with the highly elevated levels of adenosine that occur in hypoxia. Preconditioning can also be induced by exogenous AR agonists, potentially through activation of each of the four AR subtypes, which are present on cardiac myocytes [34,35]. Adenosine pretreatment also conditions against ischemic cardiac damage ~24–72 h later [36]. The cardioprotection by selective opioid receptor agonists, such as natrindole (δ-opioid) or GNTI (κ-opioid), in remote preconditioning was found to be dependent on AR signaling (using 10 µM **26**, 1 µM **39**, and 100 µM **24**), possibly through a direct interaction between the opioid receptors and the A_1_AR [37]. Sildenafil **51**, an inhibitor of PDE5 that was originally developed as an antihypertensive agent and is used for treating erectile dysfunction, induces a delayed cardioprotective effect. This protection in the mouse heart is absent in A_1_AR KO mice or upon treatment with the A_1_AR antagonist DPCPX **26** (0.1 mg/kg, i.p.) [38], suggesting that sildenafil activates adenosinergic signaling by an unclear mechanism. The anti-ischemic effect in the heart of anti-angina drug ranolazine **61**, a sodium and potassium channel blocker, appears be dependent on it causing elevated adenosine levels, which would account for its anti-adrenergic and cardioprotective effects, possibly by inhibition of cytosolic 5′-nucleotidase [39].

Metformin **49** is a first-line drug for treating type 2 diabetes mellitus (T2D) and is in clinical trials for other indications [40]. Metformin likely affects the cellular energy state, but the specific mechanism(s) involved are debated. Elucidating metformin’s molecular target(s) has been hampered by the high doses and tissue/cell concentrations required for clinical efficacy. Proposed mechanisms, which are not mutually exclusive, include inhibition of respiratory chain complex 1, inhibition of mitochondrial glycerophosphate dehydrogenase, activation of AMPK, and effects in the intestine (both microbiome-dependent and independent) [41]. Metformin also inhibits AMP deaminase [42,43], but at concentrations higher than are achieved clinically. The proposal that metformin can increase extracellular adenosine by inhibiting adenosine kinase was not supported by direct experiments [44].

Nicotinamide riboside (NR) **53**, a metabolic precursor of NAD^+^, has anti-aging effects and provides health benefits that mimic dietary calorie restriction [45]. It is widely available as a dietary supplement. NR increased ATP and its metabolites ADP and adenosine. Adenosine content in peripheral blood mononuclear cells from human subjects was raised by 50%, following dietary NR supplementation, compared to a placebo.

The antiplatelet drug ticagrelor **40** is principally a reversible inhibitor of the ADP-activated P2Y_12_ receptor. It has a secondary action that causes an increase in extracellular adenosine by blocking the ENT1 in erythrocytes in whole blood and endothelial cells [46]. The adenosine is thought to act on the A_2A_AR on platelets to contribute to ticagrelor’s desired antiaggregatory effect (antagonized by 14 µM **32**) and is possibly implicated in the drug’s side effect of dyspnea [46,47]. Nimodipine **41**, an L-type Ca^2+^ channel blocker used for treating vasospasm, at pharmacological doses inhibits ENT1 binding and adenosine uptake [48]. [^3^H](±)Nimodipine bound to the nucleoside transporter in human red blood cell ghosts with a potent K_d_ of 52 nM. The (+)-isomer of nimodipine was preferred at the transporter, in contrast to Ca^2+^ channels, which prefer the (-)-isomer. Binding affinities of several other dihydropyridine Ca^2+^ channel blockers were weaker, yet measurable. Mineralocorticoid receptor antagonist canrenone (a steroid derivative, structure not shown) has been proposed to exert cardioprotective effects through A_2B_AR activation as a result of increased extracellular adenosine [49]). Eplerenone (structure not shown), also an antagonist of the cytosolic mineralocorticoid receptor, is cardioprotective, and its benefit was lost when adenosine signaling was blocked in mice and rats [50]. However, administration of eplerenone in healthy human subjects did not elevate circulating adenosine levels [50]. Thus, approved drugs of various classes have been found to inhibit adenosine transporters in vitro, and, in some cases, elevated adenosine might contribute to their in vivo cardiovascular effects.

The widely used anti-claudication drug cilostazol **44** acts as a dual inhibitor of PDE3 and adenosine uptake [51,52]. Cilostazol-induced cardioprotection is antagonized by a non-selective AR antagonist 8-sulfophenyl-theophylline **24** (7.5 mg/kg, i.v.) [53]. Chronic administration of the immunosuppressive drug cyclosporin A (structure not shown) blocks ENT uptake of adenosine to increase its plasma levels [54]. The binding K_i_ values of known ENT1 inhibitors, such as the potent draflazine (structure not shown), and their IC_50_ values in adenosine uptake were compared [55], and the ENT1 potency correlated with bronchospasm and dyspnea in patients with respiratory conditions. In screening 1625 diverse drug molecules, 22% displayed K_i_ values <1 µM and 58% had K_i_ values <10 µM, indicating that ENT1 is a relatively common off-target activity and requires careful evaluation in the drug development process. In addition to compounds that block update by binding to transporters, some compounds regulate levels of ectonucleotidases. Cholesterol-lowering statin drugs enhance CD73 expression in cardiomyocytes and endothelial cells; administration of rosuvastatin (structure not shown) in humans augmented adenosine produced an additive vasodilation with dipyridamole and protection against ischemia-reperfusion injury, which were antagonized by the pan-AR antagonist caffeine [56].

### 2.2. Treatment of Inflammation

The adenosinergic mechanisms of three classes of anti-inflammatory drugs have been extensively explored by Cronstein and colleagues [57]. Low-dose MTX, a folate antimetabolite which is used in cancer treatment at 100-fold higher doses, is a classic treatment of rheumatoid arthritis. Cronstein and coworkers found that its MoA involves increasing adenosine levels, which results in the activation of the A_2A_AR on immune cells [9]. Human polymorphisms of the adenosine monophosphate deaminase 1 (AMPD1), inosine monophosphate synthase (ATIC), and inosine triphosphate pyrophosphohydrolase (ITPA) genes that are involved in adenosine release correlate with favorable responses to MTX treatment for rheumatoid arthritis [58]. The polyglutamated form of MTX indirectly increases levels of 5-aminoimidazole-4-carboxamide riboside (**50**, AICA riboside, AICAR), by inhibiting its metabolism by AICAR transformylase [9]. Increased intracellular AICAR results in higher adenosine levels (see below). In the in vivo mouse air-pouch model of inflammation, methotrexate increased levels of AICAR in spleen cells, and in exudates increased adenosine to inhibit leukocyte accumulation. Moreover, **22** and **23** in rats (10 mg/kg/day) reversed anti-inflammatory effects of MTX [9]. Thus, modulation of adenosine levels by MTX is a plausible mechanism that could contribute to its clinical anti-inflammatory efficacy.

The MoA in the periphery of acetylsalicylic acid (ASA), a non-steroidal anti-inflammatory drug (NSAID) and a prodrug of salicylic acid **48**, is thought to involve in part ARs [57]. Previously, high doses of ASA (3–5 g/day) administered to rheumatoid arthritis patients [59] were shown to elevate adenosine signaling. Such high ASA doses are no longer standard treatment. However, in the CNS, the expression of proinflammatory COX-2 was upregulated by A_2A_AR activation, suggesting the use of an A_2A_AR antagonist for reducing neuroinflammation [60]. Although the principal MoA of ASA is to inhibit cyclooxygenase (COX1 and 2) enzymes, its chemical precursor salicylate acts via multiple pathways. It decreases leukocyte counts, likely through effects on nuclear factor-κB, while also inhibiting various dehydrogenases and kinases, stimulating AMPK, and improving glycemia in T2D patients [61]. Salicylate also uncouples oxidative phosphorylation, leading to increased intracellular AMP and, indirectly, extracellular adenosine [57]. The anti-inflammatory drug sulfasalazine **54** is a prodrug of 5-amino-salicylic acid and is used in rheumatoid arthritis patients that do not respond to NSAIDs. Unlike salicylic acid, it is considered a disease-modifying antirheumatic drug (DMARD) by suppressing immune system function. There is evidence that the MoA of sulfasalazine also involves adenosinergic signaling by interfering with AICAR metabolism (antagonized by 0.5 mg/kg **29** in murine air pouch) [57,62]. Another NSAID, the anti-inflammatory drug sulindac **45**, has an active metabolite, sulindac sulfide, that competitively inhibits ENT1 [12] (Li et al., 2012). It inhibits adenosine uptake in human aortic smooth muscle cells with an IC_50_ (~40 µM) of roughly twice its peak therapeutic plasma concentration, while other NSAIDs were shown to be inactive or only weakly inhibiting adenosine uptake. Thus, certain NSAIDs at high therapeutic doses can modulate adenosinergic signaling. 

Glucocorticoids are foundational anti-inflammatory drugs. Cronstein and colleagues did not find an adenosine-dependent MoA for the action of glucocorticoids on leukocytes [57]. However, there is a report suggesting that use of glucocorticoids in treating inflammation involves ARs as a secondary mechanism, i.e., activation of the A_3_AR (antagonized by 0.5 µM **38**) to promote survival of anti-inflammatory human monocytes [63]. The anti-inflammatory effect of ketamine **47**, an N-methyl-D-aspartatic acid (NMDA) receptor antagonist (see Section 2.3.), has been ascribed to ARs (in LPS-induced mouse sepsis, antagonized by 1 mg/kg **32** but not 10 mg/kg **26**, i.p.) [64]. Macrocyclic derivatives related to the immunosuppressant drug rapamycin were found to block adenosine uptake by binding to ENT1 and consequently increase AR signaling [65]. 

There may be a connection between ARs and the anti-inflammatory effects of the cannabinoid system. The cannabinoid metabolite cannabidiol (**42**, CBD) was recently approved by the FDA for rare forms of childhood epilepsy. CDB does not bind potently to cannabinoid receptors (CBRs) or to ARs, but it inhibits adenosine uptake by binding to ENT1 with a K_i_ value of <250 nM [66], which may contribute to its anti-inflammatory effect by indirectly activating the A_2A_AR [67]. The anti-ischemic and anti-stroke activity of CBD partly results from AR activation (CBD reduction of glutamate release antagonized by 100 µM **23**) [68]. In newborn mouse brain slices subjected to deprivation of oxygen and glucose, CDB (100 µM) reduced hypoxic ischemic brain damage by lowering glutamate release (associated with A_1_AR activation) [5], and expression of IL-6, TNFα, COX-2, and iNOS (all reversed by A_2A_AR or CB_2_R antagonists at high concentrations). A_1_AR in the brain suppresses the release of excitatory neurotransmitters [31,69]. CBD-dependent decrease of inflammation in acute lung injury (antagonized by 5 mg/kg **32**, i.p.) and in the retina (antagonized by 500 nM **32** but not 100 nM **23**) is also dependent on the A_2A_AR [70,71]. Pretreatment of mice with 20 mg/kg CBD (i.p.) 60 min prior to lipopolysaccharide-induced lung injury reduced neutrophil migration and induction of pro-inflammatory cytokines, and these effects were antagonized by the A_2A_AR antagonist ZM241385 **32** (5 mg/kg, i.p.) administered 30 min prior to CBD. DMH-CBD **43**, a dimethyl, chain-extended analogue of CBD was found to reduce NFκB activity by indirectly activating the A_2A_AR [72]. The adenosinergic mechanisms proposed for CBD and its analogues merit further validation, using AR KO mice.

### 2.3. Pain, Antidepressant, Sleep, and Other Behavioral Intervention

Activation of each of the AR subtypes, except A_2B_AR [73], has been found to relieve pain in various animal models [74]. For decades, a connection between the opioid system and the adenosine system has been known [10]. Morphine induces the release of adenosine to activate the A_1_AR in the spinal cord (shown in synaptosomes using 10 µM **21**) [75], an effect that is attenuated in chronic neuropathic pain [76].

A_1_AR activation has also been shown to produce an antidepressant effect that mimics the effect of sleep deprivation, which increases brain adenosine [77]. The antidepressant effect of A_1_AR activation is mediated by the transcription factor homer1a in the brain, which was demonstrated by using both AR agonists and A_1_AR KO mice [78].

The anesthetic drug and NMDA receptor uncompetitive inhibitor ketamine **47** is a novel antidepressant medication having a sustained effect with rapid onset. Its antidepressant action, as evident in the mouse tail suspension assay, is dependent on activation of A_1_AR and A_2A_AR (using **23**, **26**, and **32** at 3, 2, and 1 mg/kg, i.p., respectively) [79]. The antidepressant effects of ketamine and creatine **56**, a metabolite that facilitates ATP recycling, were reduced by AR antagonists caffeine **23**, DPCPX **26,** and ZM241385 **32** and produced synergistic antidepressant effects with co-administered AR agonists. The anxiolytic effect of another NMDA receptor antagonist, dizocilpine (**55**, MK801, 50 µg/kg), on mice in the elevated plus-maze depends on A_1_AR activation by endogenous adenosine, as determined by coadministration of DPCPX **26** (50 µg/kg, i.p.) [80]. Thus, the antidepressant or anxiolytic effects of some approved treatments can have an endogenous adenosinergic component in their MoA, consistent with experiments with exogenous AR agonists [78].

Amitriptyline **46** is an inhibitor of norepinephrine and other neurotransmitter transporters that is used to treat depression and chronic neuropathic pain. Increased A_1_AR signaling in the spine and periphery has been proposed as a MoA of amitriptyline, along with 5HT_7_ receptor agonism secondarily [81]. Amitriptyline (10 mg/kg, i.p.) was also found to increase the pain threshold of neuropathic rats following sciatic nerve ligation and reduce the associated proinflammatory signaling to ERK1/2 and CREB [82]. Both effects of amitriptyline were antagonized by A_3_AR antagonist MRS1191 **36** (1 mg/kg subcutaneously immediately before amitriptyline). However, whether the A_1_AR- and A_3_AR-dependent effects of amitriptyline were the result of increased adenosine levels or the mechanisms involved was not probed. Moreover, amitriptyline binds with moderate affinity to human A_2A_AR (K_i_ 4.8 ± 0.11 μM), but not to A_1_AR, which might contribute to its side effects [83]. 

Gabapentin **52,** an inhibitor of α_2_δ subunits of voltage-dependent calcium channels, is widely used for treating seizures and chronic neuropathic pain. Its anti-hyperalgesic affect in mice was antagonized by the nonselective AR antagonist caffeine and by intrathecal administration of the selective A_1_AR antagonist DPCPX **26** (3 µg) [84]. Thus, its beneficial action in pain may be partly dependent on A_1_AR activation. Gabapentin is also used to treat restless leg syndrome, which was recently reported to have a MoA involving the A_1_AR [85]. One of the more unusual proposals is a peripheral adenosinergic MoA of warm-water-immersion therapy for treating persistent inflammatory pain, based on the antagonism by locally administered DPCPX (10 nmol/paw) [86].

The experimental anti-ischemic and anticancer drug AICAR enters the cell through nucleoside transporters ENT1 and CNT3 [87], and, as its 5′-monophosphorylated product ZMP, activates AMPK. AICAR also competes with adenosine in nucleoside transport, which contributes to elevated extracellular adenosine and depression of excitatory synaptic transmission (antagonized by 1 µM cyclopentyltheophylline) in the rat hippocampus [88]. AICAR also raises coronary blood levels of adenosine during heart ischemia [6].

The neuropeptide orexin, which binds to two orexin receptor GPCRs, induces an antinociceptive effect that is measurable in a rat model of colonic distension. Unlike other models in which adenosine has an antinociceptive effect, in this model A_1_AR signaling (antagonized using **26**, 1 mg/kg, s.c.) increases nociception [89]. Thus, an A_1_AR antagonist is predicted to be beneficial in this context. Note that non-selective AR antagonist caffeine is used in combination with over-the-counter pain medications to boost their effectiveness, but the reasons are unclear. Orexin also has a dual role as enhancer and suppressive compensator in central A_1_AR-induced hypothermia [90].

Various herbal pain treatments are thought to involve AR signaling in mouse (*Haematostaphis barteri*, using 5 mg/kg **22**, p.o. [91]; *Clinacanthus nutans*, using 3 mg/kg **23**, i.p. [92]). An antinociceptive and neuroprotective traditional Chinese medicine containing paeoniflorin, a monoterpene glucoside isolated from peony root, has allosteric A_1_AR activation as its putative mechanism of action, but pharmacologically distinct from **16** [93]. Uliginosin B is a naturally occurring acylphloroglucinol that reduces pain, and its MoA appears to involve adenosine signaling [94]. Incarvillateine is a complex monoterpene alkaloid that induces an antinociceptive effect, which is associated with AR signaling (using **26** and **29** at 0.1 and 1 mg/kg, i.p., respectively), but not opioid receptor activation [95]. Thus, various traditional medicines may in fact work through an adenosinergic MoA.

The pain-suppressing effect of acupuncture was found to be dependent on A_1_AR signaling. The proposed mechanism is that an acupuncture needle increases transient, local levels of ATP, itself a pronociceptive agent, which indirectly raises adenosine levels to activate the A_1_AR on peripheral sensory nerves [96]. ATP acting at the P2X3 receptor is known to mediate the painful effects of distension [19], and its hydrolysis product adenosine would reduce pain. Furthermore, electroacupuncture is effective in suppressing inflammation in part by A_1_AR signaling (antagonized by A_1_AR selective rolofylline (structure not shown), 3 mg/kg, i.m.) [97].

The main MoA of the benzodiazepines as anti-anxiety drugs is as allosteric activators of the GABA_A_ receptor, but other factors influence its action. Benzodiazepine anxiolytics were reported by Phillis and Wu [8] as among many diverse, centrally active drugs to inhibit adenosine uptake, usually at micromolar concentrations, along with antipsychotics trifluoperazine, spiroperidol, and sulpiride. Like gabapentin, benzodiazepines are also used to treat restless leg syndrome, a condition that is now associated with the A_1_AR [85]. Curiously, some indirect effects of the benzodiazepine diazepam **53** on this receptor have been demonstrated, consistent with diazepam reversing some of the stimulant effects of caffeine. However, diazepam decreases A_2B_AR signaling, though it does not bind directly to the receptor [98]. Chronic diazepam treatment lowered the density of hippocampal A_1_AR and striatal A_2A_AR radioligand binding in the brain by 13% and 46%, respectively [99]. However, in vivo brain binding of [^3^H]DPCPX **26** (A_1_AR antagonist) was decreased by chronic exposure of mice to two other benzodiazepines [100].

The antidepressant drug sertraline **58**, a selective serotonin reuptake inhibitor (SSRI), reduced the level of ADA activity in rats [101]). Tianeptine **57**, an atypical tricyclic antidepressant, delayed the onset time of seizures induced by pentylenetetrazole in mice. A mechanism of action of tianeptine might be indirect A_1_AR activation, based on inhibition of the protection by DPCPX **26** (20 mg/kg, i.p.), but not CSC (8 mg/kg, i.p.) [102]. Zinc salts are used as dietary supplements for treating the common cold and other conditions. Zinc chloride (30 mg/kg, i.p.) induced an antidepressant effect in the mouse forced swimming test that was blocked by caffeine or antagonists of A_1_AR (DPCPX **26**, 2 mg/kg i.p.) or A_2A_AR (ZM241385 **32**, 1 mg/kg i.p.) [103]. Somewhat selective agonists of those AR subtypes and dipyridamole **21** potentiated the effect of a subthreshold dose of Zn^2+^.

Adenosine acts through the A_1_AR and A_2A_AR in the brain, to promote sleep. Melatonin receptors are involved in regulation of circadian rhythm and the sleep cycle, based partly on larval exposure to **3** and **18** (50 and 30 µM, respectively) [104]. Gandhi et al. [105] found that melatonin **60** promotes sleep in part through Ars, using A_2A_AR KO mice and ADK inhibition with ABT-702 **18**. Melatonin promotes the local release of forebrain adenosine in mammals. The involvement of adenosine could provide a mechanistic link between circadian and homeostatic sleep control.

One of the effects of ethanol is the inhibition of adenosine uptake through transporters (ENT1), which raises the level of extracellular adenosine [106]. With acute (but not chronic) exposure to clinically relevant ethanol concentrations of 50–200 mM, the uptake of adenosine in lymphoma cells was decreased by 30–40%, leading to increased extracellular adenosine. Using A_2A_AR KO mice or following pretreatment of WT mice with nonselective antagonist caffeine, A_2A_AR was found to mediate the hypnotic effects of ethanol indicated by NREM sleep (blocked by 10 mg/kg **23**, i.p., and in A_2A_AR KO mouse) [107]. Acute ethanol exposure has a dual effect on adenosine signaling in rat hippocampal slices [108]. Depressant concentrations of ethanol rapidly increased the basal release of adenosine (leading to A_1_AR activation), but its release was inhibited during electrographic seizure activity that was induced by using convulsant drug 4-aminopyridine. The known hypothermic effect of ethanol was partly blunted in A_2A_AR KO mice, in both males and females [109] (Naasila et al., 2002). A_1_AR activation is also involved in the sedative/hypnotic effects of ethanol and is a primary mechanism of ethanol-induced ataxia [31]. ENT1-null mice have a decreased adenosine tone, a reduced response to ethanol, and greater ethanol consumption [110].

### 2.4. Anticancer Drugs

The anticancer drug deoxycoformycin (pentostatin, DCF **20**) inhibits intracellular ADA to interfere with DNA synthesis. A secondary consequence of ADA inhibition is a rise in extracellular adenosine, which can activate ARs. Several ADA inhibitors, including DCF, were shown to moderately raise the concentration of extracellular adenosine in guinea pig atria to augment exogenous agonist-induced myocardial A_1_AR activation [111]. Similar effects of DCF to raise endogenous adenosine levels in vivo were reported earlier. Conversely, drugs that block the formation or action of adenosine are experimental drugs in cancer immunotherapy, as elevated adenosine in the tumor microenvironment is immunosuppressive [1,16].

The cell entry and cytotoxicity of anticancer nucleosides such as cytarabine, fludarabine, and gemcitabine is dependent on nucleoside transporters, and transporter levels in cancer cells can be a determinant of drug efficacy and toxicity [13]. However, the transporters can be downregulated to create drug resistance [7]. Non-nucleoside anticancer drug gefitinib and other tyrosine kinase inhibitors also inhibit nucleoside transporters.

### 2.5. AR Interaction with Other GPCRs

ARs can heterodimerize with other GPCRs, as shown for A_1_AR and the dopamine D_1_R [112]. These direct GPCR interactions enable allosteric pharmacological interactions to occur. For example, disruption of the A_2A_AR–D_2_R complex by an A_2A_AR agonist blocks the inhibition of cocaine self-administration [113]. Thus, there can potentially be MoA interactions between ARs and any of the numerous other GPCRs with which they heterodimerize or oligomerize (reviewed in Vecchio et al. [114,115]). Interactions of A_2A_AR–mGlu_5_R (metabotropic glutamate receptor) are thought to be important in Parkinson’s disease. Thus, non-adenosinergic agents can interact with purinergic signaling pathways through the allosteric interactions within GPCR heterodimers.

Adenosine was reported to be a partial agonist of the ghrelin receptor, GHSR1a, without a documented requirement for heterodimerization [116]. However, a subsequent report questioned this mechanism [117].

### 2.6. Other Unanticipated Interactions with ARs

Off-target binding at one or more of the ARs has been detected for various drugs, suggesting a direct interaction, typically as an antagonist. In some cases, an off-target interaction might either reinforce or attenuate the benefit from the drug treatment. Mefloquine **66**, an antimalarial drug that displays significant CNS side effects, was found to be a moderately potent A_1_AR and A_2A_AR antagonist, with K_i_ values of the (-)-(*R*,*S*)-isomer of 255 and 61 nM, respectively. This led to the design of additional analogues with increased A_2A_AR affinity as potential anti-Parkinson’s drugs [118]. The AR antagonism and other off-target GPCR activity might contribute to mefloquine’s side effects. Dipyridamole **21** (K_i_ = 19 µM), proadifen (49 µM), a cytochrome P450 monooxygenase inhibitor, and folic acid (28 µM) weakly inhibit radioligand binding at the rat A_3_AR [119]. Cardiotonic drug sulmazole **65** (23 µM), anti-asthmatic zindotrine **63** (0.9 µM), and proconvulsant beta-carboline DMCM **64** (3.3 µM) inhibit radioligand binding at the rat A_2A_AR [119]. Sulmazole **65** (11 µM) and DMCM (1.6 µM) also inhibit rat A_1_AR binding [119,120]. A_3_AR binding is inhibited by other drug substances, identified using high-throughput fluorescent screening of a chemical library: K114 (**67**, K_i_ 372 nM), Src inhibitor SU6656 (**68**, K_i_ 676 nM), and retinoic acid p-hydroxyanilide (**69**, K_i_ 741 nM, fenretinide) [121].

Some flavonoids, such as genistein, galangin, and hispidol (structures not shown), which are found in the human diet, are known to bind to A_1_AR and A_3_AR [122]. In addition, the flavone quercetin inhibits both the activity and expression of CD73, leading to a reduction of adenosinergic signaling [123]. In silico screening for binding at ARs, based on receptor X-ray structures, has identified numerous diverse chemotypes as candidate ligands, including some with known biological activity [124]. The antiviral drug ribavirin binds to the A_1_AR as a partial agonist [25].

With broad screening of drug candidates now standard, more compounds have been found to interact with purinergic signaling pathways. For example, AMG 337 (structure not shown), an experimental kinase inhibitor for cancer, was found to inhibit ENT1 at < 1 µM, and this was suggested to cause cerebral vasorelaxation and thus the dose-limiting headaches in patients [125]. 

Other nonpharmacological treatments are reported to interact with the adenosine system. Refractory seizure activity can be treated with a ketogenic diet, which works in part by increasing A_1_AR-mediated inhibition of seizure activity [126]. Electroconvulsive therapy as a treatment for mood disorders has been reported to act through the release of ATP to indirectly raise adenosine concentrations and activate the brain A_1_AR, leading to its antidepressant effect [127]. Curiously, pulsating electromagnetic fields (PEMFs) were found to upregulate adenosinergic signaling in cancer and inflammation models [128,129].

## 3. Conclusions

We have collected reports on the unanticipated involvement of adenosinergic signaling in the action of diverse drugs. Some of the compounds or treatments are thought to raise endogenous adenosine levels by inhibiting adenosine transport, e.g., ticagrelor, ethanol, and CBD. Others presumably activate one or more of the ARs by raising adenosine levels or by undetermined or unclear mechanisms, e.g., PEMFs, or through intracellular pathways, e.g., methotrexate, salicylate, NR, and AICAR. However, other compounds directly bind one or more of the ARs, e.g., mefloquine.

Most of the detected interactions have not yet been validated as a MoA in humans. Some of these reports might represent epiphenomena, and we have commented on the strength of the evidence in some cases. Some of the effects may constitute one aspect of a cluster of actions. Therefore, it is essential that these observations be validated and characterized, using modern tools, such as more specific pharmacological agents and genetically modified mouse lines. Given the interest in AR modulation for treatment of cancer, CNS, immune, metabolic, cardiovascular, and musculoskeletal conditions, it is informative to consider AR and non-AR adenosinergic effects of approved drugs and conventional treatments.

## Figures and Tables

**Table 1:** Affinities of selected AR ligands (K_i_, nM) that have been used to define adenosinergic activities of non-adenosine receptor drugs (refer to Figure 2 for most structures). Species are human (h), unless noted (m, mouse; r, rat). Values shown and for other compounds in Figure 2 are in Müller and Jacobson; Carlin et al.; Wan et al.; Tosh et al.; Alnouri et al. [22,23,24,25,26]. Values that represent >100-fold selectivity are shown in bold italics.

Compound	A_1_AR	A_2A_AR	A_2B_AR	A_3_AR
Agonists				
Adenosine **1**	~100 73 (r)	310 150 (r)	15,000 5100 (r)	290 6500 (r)
R-PIA **4**	2.04 1.2 (r)	220 (r)	150,000	33 158 (r)
CGS21680 **8**	289 193 (m)	27 10 (m)	>10,000	67 48 (m)
Regadenoson **9**	>16,000 7.75 (m)	*290* 77.2 (m)	>10,000 >100,000 (m)	>10,000 >10,000 (m)
Bay60-6583 ^a^ **12**	>10,000 351 (m)	>10,000 >10,000 (m)	*3–10* 136 (m)	>10,000 3920 (m)
IB-MECA **13**	51 5.9 (m)	2900 ~1000 (m)	11,000	1.8 0.087 (m)
Cl-IB-MECA **14**	220 35 (m)	5360 ~10,000 (m)	>10,000	61.4 *0.18 (m)*
Antagonists				
Caffeine **23**	10,700	24,300	33,800	13,300 >100,000 (r)
8-SPT **24**	537 (m)	12,400 (m)	4990 (m)	>10,000 (m)
XAC **25**	6.8 1.2 (r), 2.2 (m)	18.4 63 (r), 83 (m)	7.75 63 (r), 4.5 (m)	25.6 29,000 (r), ~10,000 (m)
DPCPX **26**	3.0 1.5 (m)	129 598 (m)	51 86.2 (m)	795 >10,000 (r, m)
CSC ^b^	28,000 (r)	54 (r)	8200 (r)	>10,000 (r)
SCH442416 **28**	1110 765 (m)	*4.1* *1.27 (m)*	>10,000	>10,000 >10,000 (m)
ZM241385 **32**	774 249 (m)	1.6 0.72 (m)	75 31 (m)	743 10,000 (m)
MRS1220 **38**	81 (m)	9.1 (m)	ND	>10,000 (m)
	305 (r)	52 (r)	ND	0.65 (h)
MRS1523 **39**	>10,000 15,600 (r) 5330 (m)	3660 2050 (r) >10,000 (m)	>10,000 >10,000 (m)	18.9 113 (r) 702 (m)

Notes: ^a^—may be antagonist at A_1_AR and A_3_AR, partial agonist at A_2B_AR; ^b^—CSC (structure not shown) has a second activity to inhibit monoamine oxidase: K_i_ MAO-B, 80.6 nM. ND, not determined.
